# Exploring methodology for investigating Chinese coronary artery disease patient values and preferences: A methodological study protocol

**DOI:** 10.1016/j.ghrp.2026.02.001

**Published:** 2026-02-11

**Authors:** Changning Liu, Gordon Guyatt, Yunxian Zhou, Hongling Chu, Linan Zeng, Ruijin Qiu, Tianqi Zuo, Fengxia Lin, Shichao Lv, Xiaoyu Zhang, Rui Zheng, Siyu Yan, Zumao Cao, Miao Qu, Gang Sun, Qinwei Fu, Hamed Movahed, Yinghui Jin, Hongcai Shang

**Affiliations:** a Dongzhimen Hospital, Beijing University of Chinese Medicine, Beijing, China; b Key Laboratory of Chinese Internal Medicine of Ministry of Education, Beijing University of Chinese Medicine, Beijing, China; c Department of Health Research Methods, Evidence, and Impact, McMaster University, Hamilton, Canada; d Department of Medicine, McMaster University, Hamilton, Canada; e MAGIC Evidence Ecosystem Foundation, Oslo, Norway; f School of Nursing, Zhejiang Chinese Medical University, Hangzhou, China; g Research Center of Clinical Epidemiology, Peking University Third Hospital, Beijing, China; h Pharmacy Department/Evidence-based Pharmacy Centre/Children's Medicine Key Laboratory of Sichuan Province, West China Second University Hospital, Sichuan University, Chengdu, China; i Dongfang Hospital, Beijing University of Chinese Medicine, Beijing, China; j Department of Cardiology, Shenzhen Bao'an Chinese Medicine Hospital, Guangzhou University of Chinese Medicine, Shenzhen, China; k First Teaching Hospital of Tianjin University of Traditional Chinese Medicine, National Clinical Research Center for Chinese Medicine Acupuncture and Moxibustion, Tianjin, China; l Institute of Clinical Basic Medicine of Traditional Chinese Medicine, China Academy of Chinese Medical Sciences, Beijing, China; m Center for Evidence-Based and Translational Medicine, Zhongnan Hospital of Wuhan University, Wuhan, China; n Neurology Department, Xuan Wu Hospital, Capital Medical University, Beijing, China; o Department of Gastroenterology and Hepatology, First Medical Center, Chinese PLA General Hospital, Beijing, China; p Hospital of Chengdu University of Traditional Chinese Medicine, Chengdu University of Traditional Chinese Medicine, Chengdu, China

**Keywords:** Minimal important difference, Patient values and preferences, Clinical practice guidelines, Coronary artery disease, Cognitive interview

## Abstract

**Background:**

Coronary artery disease (CAD) remains a leading cause of global mortality and disability. CAD patients face tradeoffs between antithrombotic therapy benefits and bleeding risks, underscoring the need to incorporate patient values and preferences into clinical guidelines. Establishing minimal important differences (MIDs) for patient-important outcomes supports clinical guideline development by determining the smallest change in outcomes that patients consider important. However, directly conducting patient surveys to establish MIDs presents several methodological challenges.

**Methods:**

We established a multidisciplinary working group to guide the MID investigation. Using a three-phase process, we identified key outcomes through literature review and discussion. We will develop draft health outcome descriptions by synthesizing evidence from clinical guidelines and qualitative studies, supplemented with patient interviews, and refine the drafts through iterative cognitive interviews. We then designed outcome-specific draft MID questionnaires and will employ cognitive interviews to assess clarity and comprehensibility.

**Discussion:**

This study will develop standard materials for surveying patient values and determining MIDs in Chinese CAD patients. The resulting methodology will support future investigations into patient-important outcomes and provide critical evidence for clinical guideline development.

## Background

Coronary artery disease (CAD) remains the leading global cause of mortality and loss of Disability Adjusted Life Years (DALYs), with myocardial infarction (MI) survivors demonstrating a 5- to 6-fold elevated risk of recurrent cardiovascular events and excess mortality compared to individuals without CAD.^1^ CAD patients require antithrombotic therapy to reduce ischemic events but face increased bleeding risk. The resulting benefit-risk tradeoff proves particularly critical for acute coronary syndrome (ACS) patients or those undergoing percutaneous coronary intervention who require dual antiplatelet therapy.^2^

Patient values and preferences refer to their health and life-related beliefs, expectations, personal inclinations, and priorities when evaluating the potential benefits, risks, burdens, and costs of different medical interventions.^3^ During clinical practice guidelines (CPGs) development, guideline panels should incorporate these values and preferences to trade off benefits of an intervention against risks, thereby determining recommendation direction and strength.^4^ The minimal important difference (MID) represents the smallest difference that people consider important, involving value judgments.^5^ Core GRADE (Grading of Recommendations Assessment, Development and Evaluation) methodology emphasizes establishing MIDs for each patient-important outcome.^4^ The MIDs critically inform judgments about effect importance and certainty of evidence rating.

Substantial MID advances exist in patient-reported outcomes.^6^ However, evidence remains scarce in binary outcomes. To address this critical gap, directly conducting patient surveys provides the optimal strategy for establishing MID estimates for outcomes of interest. However, implementing patient surveys faces several challenges. First, patients’ diverse perceptions or experiences of health outcomes may introduce substantial variability in their responses. Second, survey instruments require rigorous attention to issues of wording clarity, readability, and content accuracy to ensure both participants’ precise comprehension and reflection of actual patient perspectives. To address these challenges, we will conduct a methodological study to develop key tools for the MID patient survey and establish a standardized process. We anticipate that our results will be applicable to all Chinese CAD patients, and possibly such patients in other jurisdictions. This study will therefore yield critical methodological insights to ensure the scientific rigor and data accuracy of future patient surveys.

## Methods

To guide this investigation, we established a multidisciplinary working group. The group comprised experts and researchers in patient values and preferences and evidence-based research methodology (GG, HS, YJ, CL, LZ, XZ, RZ, ZC, TZ), experts in qualitative research (YZ, HC, RQ) and specialists in cardiology, neurology, and gastroenterology (FL, SL, MQ, GS). We also included two patient researchers with CAD: one male (ZF) and one female (XQ). Both were around 50 years old, held undergraduate degrees, and had no medical background. They contributed to the study design, providing insights based on their direct lived experiences. HS served as the project lead.

**Fig. 1:**
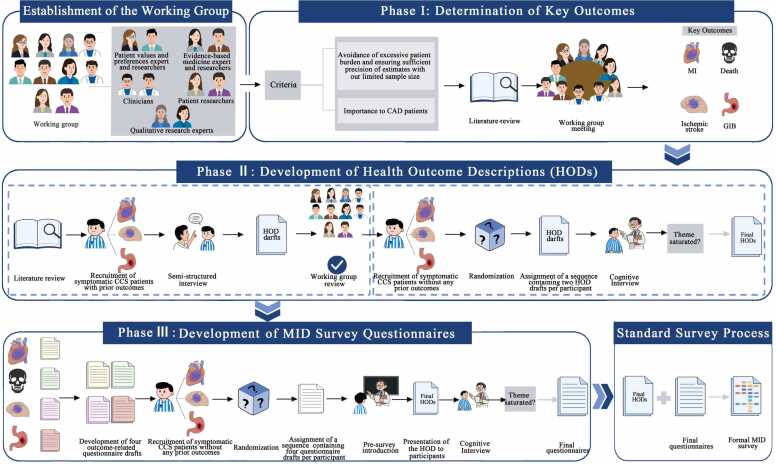
The flowchart of the study protocol.

The study will proceed through three phases. In Phase Ⅰ, which we have completed, through a literature review and discussion, the working group identified key outcomes. During Phase Ⅱ, the group will develop draft health outcome descriptions (HODs) by combining the completed literature review with semi-structured interviews, then refine and finalize them through cognitive interviews. In Phase Ⅲ, the working group has drafted questionnaires for outcomes, which will undergo cognitive interviewing. Fig. 1 illustrates the process of the investigation.

### Phase Ⅰ: determination of key outcomes

Through discussion, the working group established three criteria for selecting key desirable and undesirable outcomes: (1) avoidance of excessive patient burden and ensuring sufficient precision of estimates with our limited sample size, and (2) importance to CAD patients.

Applying the first criterion, members YJ, CL, and ZC of the working group drew on their prior MID survey experience, which showed that guideline panel members required considerable effort to complete the questionnaire and exhibited fatigue after completing the exercise with multiple outcomes. We anticipate even lower tolerance among patients. A solution to this problem would be to recruit a very large number of patients, each of whom would address only one or two outcomes. However, in terms of number of patients, our resources are limited. Therefore, acknowledging these resource constraints, and to minimize patient burden, the working group adopted random task assignment and a limited number of outcomes.

Considering the second criterion, CL and ZC conducted literature searches and identified three studies consistently reporting that CAD patients value avoiding, death, stroke, MI and bleeding events.^7^, ^8^, ^9^ Following discussion, given its relative frequency, the group selected gastrointestinal bleeding (GIB) as the representative bleeding outcome^10^, ^11^ Further, the group specified ischemic stroke due to its direct relevance to cardiovascular benefits and its use in colloquial patient communication. The working group unanimously endorsed the final four outcomes—MI, death, ischemic stroke, and GIB—as important to patients and potentially influencing treatment decisions. Appendix 1 provides further details regarding the outcome selection process.

### Phase Ⅱ: development of health outcome descriptions

The working group will develop specific descriptions for MI, ischemic stroke, and GIB. Based on input from patient researchers, the working group will refer to death simply as “death” without developing an initial HOD.

Based on prior formats and templates for presenting HODs identified in the literature,^12^, ^13^, ^14^, ^15^, ^16^ the working group identified four domains essential for inclusion in the HODs: symptoms at onset, examination and treatment, short-term consequence, and long-term consequence. Informed by previous evidence, the group will structure the HODs as bullet-points rather than narratives.^17^ To ensure participants clearly understand the outcomes they will value, the working group will develop all HODs around “typical events,” defined by two criteria: (1) clinical typicality, reflecting typical presentations and treatments aligned with CAD clinical guidelines, and (2) patient-experience typicality, capturing the symptoms and impacts commonly described by patients. These typical events will enable patients without prior experience to accurately grasp its essential features.

### Initial drafting

***Literature review:*** To ensure comprehensive and accurate HOD content and to inform the subsequent semi-structured interview guide, the researchers first conducted a literature review. They searched multiple databases to identify CPGs and qualitative studies related to MI, ischemic stroke, and GIB. From CPGs, they synthesized descriptions of typical symptoms and standard examinations and treatments. From qualitative studies, they captured patient experiences, emotional responses, and life impacts. Using a data extraction form, they categorized the extracted information into the four HOD domains. The researchers compiled all extracted content into an evidence repository for each outcome, thereby creating a systematic record of information from symptoms to consequences. Appendix 2 provides further details.

***Semi-structured interviews:*** To supplement existing evidence and ensure HODs align with Chinese patients’ experiences within their cultural context, we will conduct interviews with patients who have experienced the relevant outcomes.

Using purposive sampling with a maximum variation strategy (age, gender, educational background, marital status, and disease duration), we will recruit patients at Dongzhimen Hospital of Beijing University of Chinese Medicine. Via electronic medical records (EMRs), CL and TZ will identify potential participants. Eligible patients will be ≥ 18 years old with histories of MI, ischemic stroke, or GIB, who can provide written informed consent and possess basic literacy skills. We will cease recruitment when interviews no longer yield novel insights, i.e., reaching saturation.

Based on the evidence repositories and input from GG and YZ, CL and YJ have designed the interview guide, with a definitive version that will follow revision after pilot interviews. As an example, Table 1 shows the interview guide for the MI. Two trained interviewers will conduct the individual, face-to-face, semi-structured interviews. They will audio-record each session and transcribe the recordings verbatim within 24 h. To ensure accuracy, a third researcher will verify each transcript against the original recording. Two researchers will independently code the transcripts. They will use an initial codebook derived from the literature review and inductively create new provisional codes for data exceeding its scope, iteratively refining the codebook based on the interview data. Regular group discussion will resolve all coding discrepancies and evaluate provisional codes. This process will generate patient experience summary reports for each outcome. Each report, structured around the four HOD domains, will list codes with their frequencies and supporting patient quotations, excluding content irrelevant to the HODs.

**Table 1:** The outline of the semi-structured interview for myocardial infarction.

**Domains**	**Key Questions**
Symptom Experience	When you had your heart attack, what did it feel like in your body? Could you describe that for me in your own words?
Emotional Responses	Aside from what you felt in your body, how did the heart attack make you feel emotionally? For example, were you scared or did you feel something else?
Long-term Life Impact	Once you were back home from the hospital, did you have any chest pain or discomfort? Once back at home, did you experience any fatigue? Any shortness of breath on activity? If, back at home, you experienced any of these symptoms, how long did they go on?
	How has having a heart attack affected your day-to-day life? This could be things like your work, family duties, or your social life.

Following the summary report, researchers will draft the HODs. They will first identify high-frequency code categories. Within these categories, they will select recurring patient expressions that multiple participants used. The researchers will directly adopt these representative phrases as descriptions in the HODs. For expressions demonstrating diversity, they will synthesize all relevant quotations into concise, inclusive statements. All working group members will review the draft. When identifying medically critical information that patients rarely mentioned due to cognitive limitations, the group will supplement the draft with accessible explanations. The final draft will incorporate all working group member’s suggestions.

### Refinement of HOD drafts

***Recruitment:*** We will employ purposive sampling with a maximum variation strategy (age, gender, educational background, marital status and residence) at Dongzhimen Hospital of Beijing University of Chinese Medicine. Interviewers will identify eligible participants through EMRs.

(1) Inclusion Criteria: Age ≥18 years; meeting the diagnostic criteria for symptomatic chronic coronary syndrome (CCS) as defined in the *2024 ESC Guidelines for the Management of Chronic Coronary Syndromes*; adequate literacy and comprehension skills to complete the interview; willingness to participate and provide informed consent.
(2) Exclusion Criteria: History of MI, ischemic stroke, or GIB; presence of severe comorbidities; cognitive impairment or dementia; individuals with language or hearing barriers.

***Randomization:*** We will use an online randomization tool to assign participants without any outcome experience to receive random assignment to a first and a second specific outcome draft HODs for assessment, to achieve balance across the three HODs, a total of six allocations. If a respondent seems tired after reviewing the first HOD, the interviewer will end the session. If the interviewer observes no signs of fatigue, they will ask if the respondent wishes to complete a second assessment. If the respondent agrees, the interviewer will complete the second HOD draft evaluation to which the respondent was randomly assigned.

***Cognitive interview:*** To evaluate the clarity and comprehensibility of the draft HODs, we will engage participants in cognitive interviews. These interviews will combine “think-aloud” and “verbal probing” strategies. Participants will first review the draft HOD, verbalizing their immediate thoughts regarding the content. After putting aside the draft HOD, interviewers will ask participants to recall and restate, in their own words, key elements of the draft. The interviewers will note the retention and accuracy of the respondents’ recollection. Subsequently, guided by participants’ initial feedback, interviewers will use scripted verbal probes (see Table 2) to explore nuanced issues related to clarity, comprehension, comprehensiveness. Throughout this process, interviewers will audio-record all sessions.

**Table 2:** Cognitive interview probes for refining the HOD draft.

**Domain**	**Key Probes**
Clarify	Do you find the way that [e.g., symptoms at onset] is written clear?
Comprehension	In your own words, could you tell me what [e.g., symptoms at onset] means to you?
Comprehensiveness	What information do you think needs to be added to make it more complete and useful?
Other problems	Do you have any other questions about the description we've been discussing? Is there any way you think we could improve these descriptions?

***Data analysis:*** We will conduct multiple rounds of cognitive interviews with 7–10 participants per round, concurrently performing data collection and analysis.^18^, ^19^, ^20^ Under the guidance of qualitative research experts (HC, YZ), the researchers will analyze interview data on the website of https://wwwn.cdc.gov/QNotes.^21^ Interviewers will audio record interviews and transcribe the recordings verbatim within 24 h. To ensure accuracy, a third researcher will verify each transcript against the original recording. Following the approach suggested in the user-guide of Q-Notes, two researchers will enter the transcripts as notes into question-specific modules. They will independently code these notes, first applying deductive codes based on the four predefined probe domains, and then creating inductive codes for emergent information. They will discuss all codes and proposed revisions until reaching consensus, involving a third researcher if necessary to resolve discrepancies. The results of analysis will directly inform revisions to the draft HOD. Two field specialists will review the revised draft. Following integration of specialist feedback, interviewers will proceed to the next cognitive interview round using the updated draft HODs. Participant numbers may decrease in later rounds based on problem resolution rates as items undergo refinement. This iterative process will continue until a new round of interviews yields no further issues, indicating saturation.

### Phase Ⅲ: development of MID survey questionnaires

#### Development of draft questionnaires

Members of the working group (GG, CL, LZ, ZF and XQ) have developed four separate draft questionnaires for the MID patient survey, one for each outcome. This survey will employ face-to-face administration and present a series of outcome scenarios with varying magnitudes of intervention effect – that is, preventing adverse outcomes that will occur without treatment (MI, ischemic stroke, and death) or increasing the incidence of intervention adverse effects (GIB). For each magnitude of intervention effect, participants will independently assess the perceived importance of the decrease or increase by choosing one of four response options.

Box 1 illustrates the scenario design for MI and GIB, demonstrating how benefit/harm magnitudes and response options will be structured. Based on the prior MID survey experience of working group members (GG, LZ, YJ, CL) in guideline panels, the study preset the risk magnitude range from 1 to 50 per 1000 to ensure that most participants' choices, particularly the “small but important” rating, would fall within this predefined range.

Box 1 **Examples of questionnaire scenario design.**

**Table:** 

**Example 1: Myocardial infarction** **The first scenario states:** “*You are considering taking a medicine to prevent heart attacks. In 1000 patients, over the period of a year, this medicine reduces the occurrence of heart attack by 1. How would you rate the effect of this reduction in heart attacks?*” **The options are as follows:** − trivial, too small to be important − small but important − moderate − large In the subsequent scenarios, we will employ a "ping-pong" approach (transitioning from one extreme to the other, and gradually converging towards the center), sequentially adjusting the MI reduction to 50/1000, 5/1000, 40/1000, 10/1000, 30/1000, 15/1000, and 20/1000. The number of questions will adapt dynamically to participant responses. If participants select "small but important" or any larger effect option at low values (1/1000, 5/1000, 10/1000, 15/1000), the interviewer will terminate this phase of the interview. Similarly, if patients choose "trivial, too small to be important" at high values (20/1000, 30/1000, 40/1000, 50/1000) the interviewer will terminate this phase of the interview. **Example 2: Gastrointestinal bleeding** **The first scenario states:** *“You are considering taking a medicine to prevent blood clots. However, in 1000 patients, over the period of a year this medicine increases the occurrence of bleeding in the stomach or gut by 1. How would you rate the effect of this increase in bleeding?”* **The options are as follows:** − trivial, too small to be important − small but important − moderate − large The approach is the same as for myocardial infarction.

**Fig. 2:**
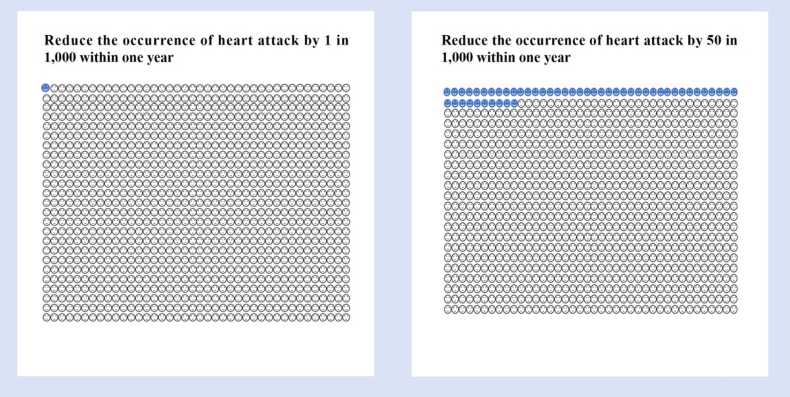
Visuals of magnitudes.

Drawing on previous research design experience,^22^, ^23^, ^24^ and considering the cognitive capacity and endurance limitations of study participants, the group developed simple, intuitive visual aids to help participants understand effect magnitudes, as shown in Fig. 2.

### Refinement of draft questionnaires

***Recruitment and allocation:*** We will replicate the approach in Phase II. Researchers will recruit participants using purposive sampling with maximum variation strategy via EMRs. The study will include consenting adults (≥18 years) with symptomatic CCS who demonstrate sufficient literacy and comprehension for interview completion. Exclusion criteria cover a history of MI, ischemic stroke, or GIB; severe comorbidities; cognitive impairment or dementia; and significant language or hearing barriers.

We first randomly ordered the four outcomes to generate 24 unique sequences. Using an online randomization tool, interviewers will assign one sequence to each participant. Upon receiving their assigned sequence, participants will complete the initial questionnaire draft. The interviewer will then ask whether the participant wishes to proceed with the second assessment. The session will end immediately if the participant reports fatigue; otherwise, they will continue to the next questionnaire draft. This process will repeat until the participant declines to continue or completes all four assessments.

***Introduction to the survey:*** Immediately prior to beginning the cognitive interview, we will engage participants in an educational session introducing the survey purpose and content, outlining questions, and explaining the rationale for the scenario design—including the risk reduction presentation “ping-pong” approach (Appendix 3).

***Presentation of final HODs:*** Following the introduction and prior to questionnaire administration, we will present participants with the final HOD of outcomes to which they’ve been allocated.

***Cognitive interview:*** We will conduct cognitive interviews to examine participants’ comprehension and accurate completion of draft questionnaires. Interviews will integrate “think-aloud” and “verbal probing”, facilitated by the Q-Notes software for systematic data capture. Through iterative cognitive interviews, we will continuously evaluate and refine the draft questionnaire’s content, risk change magnitudes, comprehensibility, and the clarity of items and response options.

Participants will verbalize their thought process in real-time while completing the questionnaire. The interviewers will subsequently use probes to explore key components of Tourangeau’s cognitive model^25^, ^26^ for survey response processes. Since the questions do not rely on retrieving real memories, we will adopt a modified version of the cognitive model to address the specific objectives and scope of this study phase, as detailed in Table 3.

**Table 3:** Cognitive interview probes for developing MID survey questionnaires.

**Domain**	**Key Probes**
Clarity	When reading this question, did any words or phrases look unclear or confusing to you?
Comprehension	In your own words, what is this question asking you to decide?
	When you read the phrase ‘reduce the occurrence of heart attack by 1 in 1000,’ what does that mean to you?
Judgement	What made you feel this reduction was [e.g., trivial/large]?
	If you wavered between ‘trivial’ and ‘small but important,’ what were you comparing?
Response	Do you think the differences between these options are clear?
	When you made a choice, did the option match what you were thinking?
Other problems	On a scale of 1–10, how hard were these questions to understand? (10 = very hard)
	Do you have any other thoughts or questions or suggestions for improving the survey?

Interviews will also gather participant feedback on the visual aids. Interviewers will specifically assess whether participants correctly interpret the graphical representation of risk magnitudes, identify any confusing elements, and suggest potential improvements. Key assessment points will include the clarity of the presented risk change magnitudes and the presence of any misleading visual cues. They will also ask participants if the visual aid lacks critical information.

In addition, we will focus on validating the appropriateness of the predefined risk change ranges and ensuring alignment between these values and participants’ perceptions of importance. This involves evaluating whether the predefined minimum, maximum, and intermediate magnitudes of risk change align with patient perceptions. We will also assess whether the eight scenarios provide sufficient steps to capture variations in patient preferences and generate reliable response distributions for MID estimation.

To mitigate cognitive load, interviewers will explicitly permit participants to pause or stop at any time. They will employ clear, concise language and verbally confirm the participant’s willingness to proceed before introducing each subsequent task. Throughout the interview, interviewers will monitor for signs of fatigue, including non-verbal cues (e.g., frequent yawning, wandering gaze, restlessness), verbal and interactional cues (e.g., markedly shorter responses, delayed reactions, requests for repetition, expressed tiredness), and task engagement cues (e.g., noticeable inattention, reluctance to continue). If they observe such signs, interviewers will first inquire about the participant’s comfort. They will end the session if fatigue persists or participants directly request to stop.

***Data analysis:*** We will replicate Phase II analytical procedures using Q-Notes, continuing iterations until achieving cognitive match and all participant-reported risk change values consistently fall within prespecified ranges.

## Discussion

This study aims to establish MIDs for key outcomes applicable to CAD patients. CAD encompasses both ACS, including MI and unstable angina, and chronic coronary syndromes (CCS), which comprises both symptomatic and asymptomatic presentations. CAD patients who have experienced an outcome such as MI will have extremely variable severity and thus variable experiences (e.g., mild versus severe MI). This variability will likely influence the value they place in avoiding the events they have experienced. To prevent prior experiences from confounding preference assessments, we will recruit patients without a history of MI, ischemic stroke, and GIB, thereby focusing on CCS population.

Within the CCS population, the study will further focus on symptomatic CCS patients. Asymptomatic patients may underestimate the importance of future health risks due to feeling well, potentially leading to lower MID estimates that do not represent the values of patients actually facing treatment decisions. In contrast, symptomatic CCS already participate in these decisions, such as choosing antithrombotic therapy. Their experience with symptoms typically fosters a more informed and relevant awareness of the disease. These two groups likely represent fundamentally different stages of disease perception and decision-making relevance. Therefore, the final study population will comprise symptomatic CCS patients who have not experienced the target outcomes. Because participants will lack experience with outcomes, the study requires HODs to ensure their full understanding. Our HODs represent typical events, and it is the value patients place on these typical events that we will address.

First, we will draft HODs using information from literature reviews and patient interviews. Interviewers will then conduct cognitive interviews to evaluate the clarity and comprehensibility of the draft HODs. They will randomly assign participants without outcome experience to evaluate two HOD drafts using an online tool with six allocation sequences to ensure balance.

Subsequently, we developed four outcome-specific draft questionnaires to explore MID values for each outcome. To assess patient understanding and ease of administration, we will conduct cognitive interviews for all questionnaire drafts. Interviewers will assign each participant one of 24 uniquely ordered outcome sequences. The evaluation will continue until the participant reports fatigue or completes all questionnaires, thereby maximizing efficiency without imposing excessive burden.

Since we will conduct this study exclusively in China, we tailored all survey materials and tools to the Chinese cultural context. This approach ensures local relevance but does not evaluate cross-cultural applicability. Future studies will need to replicate our methods in other cultural and linguistic settings to confirm broader generalizability. Nonetheless, this study will establish methodological and procedural guidance for the future large-scale MID investigations. We anticipate that this MID-based method will provide useful estimates of patient average or typical MIDs. The identification of MID for specific outcomes represents a critical step in guiding the formulation of recommendation decisions during guideline development. By determining MID for targeted outcomes, stakeholders can more effectively evaluate treatment benefits or harms within patient-centered care paradigms. The relevant Core GRADE paper describes this use of MIDs in the process of moving from evidence to recommendations.^4^

## Authors’ contributions

CL, GG, YJ, and HS conceived the study and drafted the protocol. All authors contributed to manuscript revision and approved the final submitted version.

## CRediT authorship contribution statement

**Zumao Cao:** Investigation, Data curation. **Xiaoyu Zhang:** Methodology. **Shichao Lv:** Methodology. **Hongcai Shang:** Writing – review & editing, Supervision, Resources, Methodology, Conceptualization. **Fengxia Lin:** Methodology. **Yinghui Jin:** Writing – review & editing, Supervision, Resources, Methodology, Conceptualization. **Tianqi Zuo:** Visualization, Validation, Investigation, Formal analysis, Data curation. **Hamed Movahed:** Methodology. **Ruijin Qiu:** Methodology. **Qinwei Fu:** Methodology. **Linan Zeng:** Methodology. **Gang Sun:** Methodology. **Hongling Chu:** Methodology. **Miao Qu:** Methodology. **Yunxian Zhou:** Writing – review & editing, Methodology. **Gordon Guyatt:** Writing – review & editing, Methodology, Conceptualization. **Siyu Yan:** Investigation, Data curation. **Changning Liu:** Writing – review & editing, Writing – original draft, Visualization, Validation, Methodology, Investigation, Formal analysis, Data curation, Conceptualization. **Rui Zheng:** Methodology.

## Consent for publication

Not applicable.

## Ethics approval and consent to participate

This study has received ethical approval from the Medical Ethics Committee of Dongzhimen Hospital of Beijing University of Chinese Medicine (2025DZMEC-218–02). All participants are required to provide written informed consent before participation. To acknowledge their contribution, participants will receive compensation that varies with their level of participation. Compensation will be 50 CNY for one task, 100 CNY for two tasks, and a maximum of 200 CNY for participants who complete all required tasks. The study results will be published in peer-reviewed journals and presented at national and international conferences.

## Funding

This study was supported by the Traditional Chinese Medicine inheritance and innovation “thousand million” Talents Project (Qihuang Project), and the Beijing Scholar Program.

## Declaration of Competing Interest

All authors declare no competing financial interests.

## Data Availability

No data was used for the research described in the article.

## Footnotes

Appendix A Supplementary data associated with this article can be found in the online version at doi:10.1016/j.ghrp.2026.02.001
