# Supplementary material for: Exploring methodology for investigating Chinese coronary artery disease patient values and preferences: A methodological study protocol
Source: Glob Health Res Policy. 2026 Feb 11;11(1):36–42. doi: 10.1016/j.ghrp.2026.02.001 (PMC13068842; doi:10.1016/j.ghrp.2026.02.001)
Supplement: Supplementary file 1 — Supplementary material [file mmc1.docx]

**Appendix 1 Determination of key outcomes**

Through discussions, the working group established two criteria for selecting key desirable and undesirable outcomes: (1) avoidance of excessive patient burden and ensuring sufficient precision of estimates with our limited sample size, and (2) importance to CAD patients.

To apply the first criterion, the working group drew on previous experience of members YJ, CL, and ZC in conducting an MID survey with a guideline panel. They distributed a questionnaire addressing inferences regarding what outcomes patients consider most important to all 46 panel members, including experts in evidence-based medicine, clinicians, and pharmacologists. The questionnaire covered five outcomes with eight questions each, and required each member to complete all sections. After providing pre-survey education to ensure understanding of the questionnaire, YJ, CL, and ZC observed that panel members expended considerable time and effort comprehending the questions. Furthermore, during the completion process, most panelists began showing signs of fatigue by the fourth or fifth outcome. Since these specialized professionals demonstrated limited tolerance and substantial cognitive effort was required, the working group reasoned that patient tolerance would likely be lower. A solution to this problem would be to recruit a very large number of patients, each of whom would address only one or two outcomes. However, in terms of number of patients, our resources are limited. Therefore, acknowledging these resource constraints, and to minimize patient burden, the working group adopted random task assignment and a limited number of outcomes.

To address the second criterion, CL and ZC conducted literature searches in PubMed, Web of Science, Embase, Cochrane, and CNKI. Table 1 presents the search strategy. The search aimed to identify and collect evidence regarding which specific outcomes are important to patients.

**Table 1 Search strategy for determining outcomes**

| Search No. | Search Terms |
| --- | --- |
| #1 | “coronary artery disease” [Title/Abstract] OR “Coronary Disease” [MeSH] OR “coronary heart disease” [Title/Abstract] OR “percutaneous coronary intervention” [Title/Abstract] OR CAD [Title/Abstract] OR CHD [Title/Abstract] OR PCI [Title/Abstract] OR “Myocardial Infarction” [MeSH] OR “heart attack” [Title/Abstract] OR “acute coronary syndrome” [Title/Abstract] OR “acute coronary syndrome”[MeSH] OR “MI” [Title/Abstract] OR “ACS” [Title/Abstract] OR “CCS” [Title/Abstract] OR “chronic coronary syndrome” [Title/Abstract] OR “coronary syndrome” [Title/Abstract] OR “cardiovascular disease*”[Title/Abstract] |
| #2 | “patient important outcome*” [Title/Abstract] OR “relative importance of outcome*” [Title/Abstract] OR outcome* [Title/Abstract] OR endpoint* [Title/Abstract] OR “core outcome set*” [Title/Abstract] OR COS [Title/Abstract] OR “patient centered outcome” [Title/Abstract] |
| #3 | “patient value* and preference*” [Title/Abstract] OR patient value* [Title/Abstract] OR value* [Title/Abstract] OR preference* [Title/Abstract] OR choice* [Title/Abstract] OR expectation* [Title/Abstract] OR view* [Title/Abstract] OR attitude* [Title/Abstract] OR knowledge* [Title/Abstract] OR accepta* [Title/Abstract] OR opinion* [Title/Abstract] OR perspective* [Title/Abstract] OR perception* [Title/Abstract] OR utility* [Title/Abstract] |
| #4 | #1 AND (#2 OR #3) |

They included studies related to CAD that reported values and preferences or important outcomes/endpoints, while excluding non-English, non-Chinese publications and studies without full-text availability. Finally, they identified three references, with detailed characteristics provided in Table 2. All studies consistently supported that CAD patients value and consider, death, stroke, myocardial infarction (MI) and bleeding events as important.

**Table 2 Details on the literature related to patients' values and preferences**

| First Author | Study Objective | Study Population | Research Method | Key Findings |
| --- | --- | --- | --- | --- |
| Zhong Yuan | To quantify preferences of US ACS patients and physicians for outcomes related to antithrombotic therapies (death, nonfatal strokes of varying severity, nonfatal MI, and nonfatal bleeding of varying severity) | 206 patients hospitalized for MI in the past 5 years and using aspirin or prescription antithrombotic therapy; 273 cardiologists | Online Best-Worst Scaling survey | ACS patients considered death and nonfatal major disabling stroke as the most important outcomes to avoid, with nearly equal priority. These were followed by nonfatal moderate stroke, nonfatal major bleeding requiring transfusion and nonfatal MI |
| Joshua M. Stolker | To quantify the relative importance of five common endpoints (death, MI, stroke, coronary revascularization, hospitalization for angina) between cardiovascular patients and trialists | 785 cardiovascular disease (CVD) patients and 164 clinical trialists | Weighting survey | Patients valued MI and stroke as equal to or more important than death, while clinical trialists placed greater emphasis on avoiding death. Both groups considered coronary revascularization and hospitalization for angina far less severe than death |
| Mehdi Najafzadeh | To compare preferences for anticoagulation therapy outcomes (nonfatal stroke, nonfatal MI, cardiovascular death, bleeding of varying severity) between the general population and CVD patients in the US | 284 CVD patients and 284 general population individuals | Discrete choice experiment | CVD patients valued fatal bleeding most among anticoagulation outcomes, followed by nonfatal MI, nonfatal stroke, cardiovascular death, major bleeding, and minor bleeding |

At a working group meeting, CL presented the literature review findings and proposed outcomes, prompting discussion among the members. For bleeding events, these three studies all used composite bleeding outcomes without specifying individual events. The working group selected gastrointestinal bleeding (GIB) as the key undesirable outcome, as it represents the majority of bleeding events associated with antithrombotic therapy in CAD.

Regarding stroke, the clinicians and patient researchers in the working group emphasized the need to specifically define stroke as ischemic in origin. This specification was necessary for two reasons: first, only a reduction in ischemic stroke—not hemorrhagic stroke—constitutes a cardiovascular benefit (desirable outcome). Second, in China, the general term “stroke” is seldom used in lay discourse. Instead, clinicians, patients and the public tend to use more specific terms such as “cerebral infarction”, reflecting a consistent distinction between ischemic and hemorrhagic events. Moreover, the term “stroke” itself carries ambiguous connotations in Chinese and is often misinterpreted by patients as facial paralysis. Thus, clarifying “ischemic stroke” can avoid misunderstanding and aligns with both clinical accuracy and public communication norms. Following discussion, the group finalized four key outcomes: MI, death, ischemic stroke, and GIB. All working group members affirmed that these are patient-important outcomes that could influence treatment decisions.

**Appendix 2 Literature review of the HOD initial drafting**

Following discussion, the working group decided to describe the HOD content using a typical presentation that avoids uncertain expressions or ambiguous language. The group believed this approach would better facilitate patient value judgement.

To obtain information for populating the four domains of the HODs, CL and TZ conducted literature searches in databases including PubMed, Web of Science, Embase, Cochrane, and CNKI. Table 1 details the complete search strategy.

**Table 1 Search strategy for drafting HOD**

| Search No. | Search Terms |
| --- | --- |
| #1 | “myocardial infarction” [MeSH Terms] OR “heart attack” [Title/Abstract] OR “acute coronary syndrome” [Title/Abstract] OR “acute coronary syndrome” [MeSH Terms] OR “MI” [Title/Abstract] OR “ACS” [Title/Abstract] |
| #2 | “stroke" [Title/Abstract] OR “stroke” [MeSH Terms] OR “ischemic stroke" [Title/Abstract] OR “ischemic stroke” [MeSH Terms] OR “cerebral infarction" [Title/Abstract] OR “cerebral infarction” [MeSH Terms] |
| #3 | "gastrointestinal bleeding” [Title/Abstract] OR “Gastrointestinal Hemorrhage” [MeSH Terms] OR “gastrointestinal hemorrhage” [Title/Abstract] OR “upper gastrointestinal bleeding” [Title/Abstract] OR “lower gastrointestinal hemorrhage” [Title/Abstract] OR “upper gastrointestinal hemorrhage” [Title/Abstract] OR “lower gastrointestinal bleeding” [Title/Abstract] OR “GIB” [Title/Abstract] OR “GI bleeding” [Title/Abstract] |
| #4 | "experience*" [Title/Abstract] OR "perception*" [Title/Abstract] OR perception [MeSH Terms] OR "symptom*" [Title/Abstract] OR "Consequence*" [Title/Abstract] OR "recovery" [Title/Abstract] OR "rehabilitation" [Title/Abstract] OR rehabilitation [MeSH Terms] |
| #5 | "qualitative study*" [Title/Abstract] OR “Qualitative Research” [MeSH Terms] OR "qualitative research*" [Title/Abstract] OR "ethnography" [Title/Abstract] OR "focus group" [Title/Abstract] OR “Focus Groups” [MeSH Terms] OR "grounded theory" [Title/Abstract] OR “grounded theory” [MeSH Terms] OR "interview*" [Title/Abstract] OR Interview [Publication Type] OR “Interviews as Topic” [MeSH Terms] OR "phenomenology" [Title/Abstract] OR "thematic analysis" [Title/Abstract] OR "descriptive qualitative study*" [Title/Abstract] OR "descriptive research*" [Title/Abstract] OR “observation” [Title/Abstract] OR “observation” [MeSH Terms] OR “qualitative content analysis” [Title/Abstract] |
| #6 | “clinical practice guideline*” [Title/Abstract] OR “Practice Guidelines as Topic” [MeSH Terms] OR “Practice Guideline” [Publication Type] OR “Practice Guidelines as Topic” [Title/Abstract] OR “Practice Guidelines as Topic” [MeSH Terms] OR “Practice Guideline” [Title/Abstract] OR CPGs [Title/Abstract] OR guideline* [Title/Abstract] OR Guideline [Publication Type] OR “Guidelines as Topic” [MeSH Terms] OR Guideline [Title/Abstract] OR “Guidelines as Topic” [Title/Abstract] OR recommendation* [Title/Abstract] OR “Health Planning Guidelines” [MeSH Terms] OR “Health Planning Guidelines” [Title/Abstract] |
| #7 | (#1 AND #4 AND #5) OR (#1 AND #6) |
| #8 | (#2 AND #4 AND #5) OR (#2 AND #6) |
| #9 | (#3 AND #4 AND #5) OR (#3 AND #6) |

Inclusion Criteria: the study population comprising patients aged ≥ 18 years with MI, ischemic stroke, or GIB. We focused on patient experiences pertaining to the “symptoms” and “consequence” domains within the HOD. Eligible study types included clinical guidelines and qualitative studies such as grounded theory, phenomenology, and ethnography. Exclusion Criteria: Non-English or non-Chinese publications; studies for which the full text is unavailable.

For clinical guidelines, CL and TZ reviewed guidelines published by recognized authoritative societies. They identified and synthesized typical symptoms associated with the outcomes, along with examination and treatment recommendations. These would directly inform the “typical symptoms” and “examination and treatment” domains of the HODs.

Qualitative studies captured patient experiences, emotional responses, and life impacts. Using MI as an example, researchers implemented the following procedure:

1. CL conducted initial screening of titles and abstracts. Two researchers (CL and TZ) then independently evaluated full-text articles, resolving discrepancies through discussion until reaching consensus. From 1,668 initially identified records, 162 articles underwent preliminary screening, with 26 studies ultimately included.
2. The researchers thoroughly reviewed each included study, recording HOD-relevant information including author, country, sample characteristics, themes/subthemes, author interpretations, direct patient quotations and corresponding HOD domains. For text segments containing HOD-relevant concepts, they applied open coding to assign descriptive codes such as “chest pain.”
3. Through collaborative discussion, the researchers refined coding results by: merging synonymous codes representing identical concepts; eliminating codes irrelevant to constructing typical HODs; and organizing remaining codes into the four HOD domains. This process yielded a structured data extraction form. They resolved discrepancies through discussion until reaching consensus.

(4) Using the updated extraction form, they analyzed subsequent literature, directly classifying information fitting existing frameworks while recording unclassifiable concepts as “pending review items.” Regular discussions determined these items' disposition through new code creation, merging into existing codes, or elimination. This iterative “analysis-deliberation-revision” cycle continued until reaching saturation, defined as three consecutive studies requiring no structural revisions to the extraction form. Table 2 is an example of the extraction form.

**Table 2 Examples of data extraction**

| Author | Country | Sample | Theme | Subtheme | Original Patient Statement | Summarized Description | Corresponding HOD Domain |
| --- | --- | --- | --- | --- | --- | --- | --- |
| Lee-Anne Gassner | Australia | 50 MI patients in CCU | The Metaphor of a Heart Attack | Sudden and Violent Onset of Symptoms | "... It felt like it’s gripping all the time but it also feels like it’s blocking you know really pulling and blocking." | Chest pain; Radiating pain | Symptoms at Onset |
|  |  |  |  |  | "... I started getting these violent pains in my back and my throat and down my arm and it was really violent then I had three attacks like that." |  |  |
|  |  |  |  | Personalized Sense of Attack from Symptoms | "It felt like somebody had just smashed my chest and gripped everything that’s in there and just squeezed and hung on, because it didn’t give up." |  |  |
|  |  |  |  |  | "... It felt like somebody had got something and rode it all over me." |  |  |

**Appendix 3 Introduction to the survey**

Mr./Ms. [Last Name], Hello!

We warmly invite you to take part in a survey about the values and preferences of patients with coronary heart disease (CAD). The aim of this questionnaire is to understand patients’ views on the benefits and risks related to treatment for CAD. Your answers will help doctors plan future treatments that better match what patients want and need.

Many patients believe that “I should follow the doctor’s advice”. You are absolutely right—trusting your doctor is a very important part of getting better. However, doctors also realize that when making decisions, it’s not only about the drug’s effects and risks—it’s also important to understand how patients themselves feel about whether a treatment’s benefits are important. The same treatment may be very worthwhile for some people, but less suitable for others. A key reason is that different patients have different views on how big benefits need to be for them to be worthwhile or important, and how much harm would make them feel they might want to avoid a treatment.

For example, for CAD patients, doctors often recommend medicines to reduce the risk of blood clots and serious problems like heart attacks. But these medicines can sometimes cause bleeding.

Some patients may think, “If it greatly lowers my chance of a heart attack, I’m willing to accept some risk of bleeding.” Others may feel, “Unless the drug’s benefit is large, I don’t want to take any extra risk of bleeding.” There is no right or wrong in this kind of judgment—it’s based on your personal point of view.

Therefore, this study is designed to learn what CAD patients like you consider an “important" treatment effect. Please note: your responses will not affect your current treatment. However, combining everyone’s answers will give doctors valuable insight, helping them make treatment decisions in the future that are both scientifically sound and more aligned with what patients value.

Here’s what you can expect in the questionnaire. You will read some hypothetical scenarios, such as:

“You are considering taking a medicine to prevent heart attacks. In 1,000 patients, over the period of a year, this medicine reduces the occurrence of heart attack by 1. How would you rate the effect of this reduction in heart attacks?”

You will then have four options to choose from:

- trivial, too small to be important

- small but important

- moderate

- large

These questions are all hypothetical. They are not related to your actual health condition, and there are no right or wrong answers. We simply want to know your honest opinion.

There are 8 questions like this in total. The only difference is that the number of people affected (reduce the occurrence of heart attack) will change. The numbers will vary—some higher, some lower—so we can more accurately find the point where most people begin to feel the effect is “important.” This isn’t meant to make it difficult, but to help us more accurately find the point where the change becomes important to you.

Your opinion is very valuable and represents the voice of patients like you. If you are ready, we can begin now. Please feel free to ask me any questions during the survey.
